# Spatial analysis of cattle and shoat population in Ethiopia: growth trend, distribution and market access

**DOI:** 10.1186/2193-1801-3-310

**Published:** 2014-06-24

**Authors:** Samson Leta, Frehiwot Mesele

**Affiliations:** Adami-Tullu Agricultural Research Center, P. O. Box 35, Ziway, Ethiopia

**Keywords:** Livestock population, GIS, Spatial distribution, Market access

## Abstract

The livestock subsector has an enormous contribution to Ethiopia’s national economy and livelihoods of many Ethiopians. The subsector contributes about 16.5% of the national Gross Domestic Product (GDP) and 35.6% of the agricultural GDP. It also contributes 15% of export earnings and 30% of agricultural employment. The livestock subsector currently support and sustain livelihoods for 80% of all rural population. The GDP of livestock related activities valued at 59 billion birr. Ethiopian livestock population trends, distribution and marketing vary considerably across space and time due to a variety of reasons. This study was aimed to assess cattle and shoat population growth trend, distribution and their access to market. Regression analysis was used to assess the cattle and shoat population growth trend and Geographic Information Systems (GIS) techniques were used to determine the spatial distribution of cattle and shoats, and their relative access to market. The data sets used are agricultural census (2001/02) and annual CSA agricultural sample survey (1995/96 to 2012/13). In the past eighteen years, the livestock population namely cattle, sheep and goat grew from 54.5 million to over 103.5 million with average annual increment of 3.4 million. The current average national cattle, sheep and goat population per km^2^ are estimated to be 71, 33 and 29 respectively (excluding Addis Ababa, Afar and Somali regions). From the total livestock population the country owns about 46% cattle, 43% sheep and 40% goats are reared within 10 km radius from major livestock market centres and all-weather roads. On the other hand, three fourth of the country’s land mass which comprises 15% of the cattle, 20% of the sheep and 21% of goat population is not accessible to market (greater than 30 km from major livestock market centres). It is found that the central highland regions account for the largest share of livestock population and also more accessible to market. Defining the spatial and temporal variations of livestock population is crucial in order to develop a sound and geographically targeted livestock development policy.

## Introduction

Naturally endowed with different agro-ecological zones and suitable environmental conditions, Ethiopia is a home for many livestock species and suitable for livestock production. Ethiopia is believed to have the largest livestock population in Africa (CSA 2013; Solomon et al. 2003; Tilahun and Schmidt 2012). An estimate indicates that the country is a home for about 54 million cattle, 25.5 million sheep and 24.06 million goats. From the total cattle population 98.95% are local breeds and the remaining are hybrid and exotic breeds. 99.8% of the sheep and nearly all goat population of the country are local breeds (CSA 2013).

The livestock subsector has an enormous contribution to Ethiopia’s national economy and livelihoods of many Ethiopians, and still promising to rally round the economic development of the country. Livestock plays vital roles in generating income to farmers, creating job opportunities, ensuring food security, providing services, contributing to asset, social, cultural and environmental values, and sustain livelihoods. The subsector contributes about 16.5% of the national Gross Domestic Product (GDP) and 35.6% of the agricultural GDP (Metaferia et al. 2011). It also contributes 15% of export earnings and 30% of agricultural employment (Behnke 2010). The livestock subsector currently support and sustain livelihoods for 80% of all rural population. The GDP of livestock related activities valued at birr 59 billion (Metaferia et al. 2011).

Despite high livestock population and existing favorable environmental conditions, the current livestock output of the country is little. This is associated with a number of complex and inter-related factors such as inadequate feed and nutrition, widespread diseases, poor genetic potential of local breeds, market problem, inefficiency of livestock development services with respect to credit, extension, marketing, and infrastructure (Benin et al. 2003; Jabbar et al. 2007; Negassa et al. 2011; Solomon et al. 2003). In Ethiopia, livestock production and markets vary substantially across space due to different reasons including topographical variations, market access, feed and water availability, and population characteristics. Studies indicate that livestock production is higher in areas nearer to the major livestock market centres. In 2007/08, more than 75% of cattle in the four major highland regions of Ethiopia were located within 5 hours travel time of a livestock market. On the other hand, the Ethiopia lowland pastoral areas which are affected by recurrent drought found to have spares livestock population (Tilahun and Schmidt 2012). However, no convincing study has been made so far to analyse the degree in which these factors hamper the production and distribution of livestock.

According to Metaferia et al. (2011), cattle, sheep and goats are the three most important livestock species that have a considerable important to the GDP of the country. Understanding the growth trend, spatial distribution and their relative access to market infrastructures of these livestock species is crucial in order to devise a feasible and geographically targeted livestock development policy. Due to the very important role the livestock subsector plays in the economy of the country, formulation of feasible and geographically targeted development plan regarding the subsector is indispensable. However, well-documented and recent information which can help to devise this kind of development plan in Ethiopia is lacking. This study attempts to fill this gap. This study uses regression analysis and Geographic Information Systems (GIS) to determine livestock population growth trend, spatial distribution and their access to market based on the data reported by the Central Statistics Authority (CSA).

## Methodology

### Description of the study area

The study was conducted in Ethiopia. Ethiopia is a landlocked country found in the horn of Africa. It is geographically located between 32° 30’-48° 00’ E and 3° 00’–15° 00’ N. It covers a land area of 1.04 million km^2^ (CIA 2014). Ethiopia is suitable for agricultural production and it is also a home for different livestock species. In 1991, when the present federal government of Ethiopia came to power, it launched Agricultural Development Led Industrialization (ADLI) strategy (Benin et al. 2003). The Ethiopian government has also formulated a five year growth and transformation plan (GTP) 2010/11 – 2014/2015 to carry forward the important strategic direction pursued over last couple of decades. In the GTP, special emphasis was given to agricultural and rural development, industry and infrastructure. The plan takes in to account two alternative economic growth scenarios. The high case scenario assumes that the GDP and the Agricultural Value Added achieved in 2009 will double by the end of the GTP period, 2014/2015 (MOFED 2010).

### Source of data

Information on the size of Ethiopian livestock populations was obtained from the CSA (http://www.csa.gov.et/) annual report series, the agricultural sample survey (CSA 1996, 1997, 1998, 1999, 2000, 2001, 2002, 2004, 2005, 2006, 2007, 2008, 2009, 2010, 2011, 2013) and agricultural census (CSA 2002). The annual Livestock Sample Survey covered the rural agricultural population in all the regions of the country except the non-sedentary population of three zones of Afar and six zones of Somali regions. The agricultural census and annual agricultural sample survey cover only three (Jijiga, Liben, and Shinile) of the nine administrative zones of the Somali region, which may not accurately represent Somali region as a whole. In order to cover the rest six zones in Somali region, an aerial survey was conducted in 2003. This aerial survey data was also used in this study.

Ethiopian major towns and roads shape file was obtained from Colorado State University (http://ethgis.colostate.edu/WebContent/WS/GISTraining/7_0_GISDataSources.html) and the Ethiopian district level administrative shape file was obtained from DIVA-GIS (http://www.diva-gis.org/).

### Methods

The national livestock growth trend was calculated using linear regression in STATA v 12 software (StataCorp. 2011). Linear regression tries to find a linear relationship between a response variable and a possible predictor variable(s). The following equation was used to fit the model

Where  is the intercept and β_j_ are coefficients of the predictor variables (X_j_).

Variable like time period (in years), the purpose of the animal, number of birth, death, slaughter, infected, treated and vaccinated were used as explanatory variable. However, numbers of animals infected, treated and vaccinated were removed from the analysis due to collinearity problem. The forecast for 2024/25 was also made using linear regression. Backward variable elimination was used to select the variables in the final model (p = 0.05) and only those variables in the final model were used to make the estimation for 2024/25.

Different GIS functionalities were used to analyze the spatial distribution of cattle and shoat population and their access to market. The agricultural sample survey is representative at the zone level and the agricultural census report of 2001/02 is representative at district level. In order to compute a district level spatial distribution of cattle and shoat, a database at district level was created using data from the agricultural census of 2001/02 (CSA 2002) and the agricultural sample survey 2012/13. The district level spatial distribution of cattle, sheep and goats for the year 2012/13 was estimated by calculating the district share of livestock population for each zone recorded in the 2001/02 census report; assuming livestock population movements, birth, death, destocking and restocking within each district in a given zone to be uniform during this time. Similar procedure was also used by Tilahun and Schmidt (2012).

Annual CSA surveys cover only two of the five zones in Afar region and three of the nine zones in Somali region, leaving out pastoral zones with high numbers of livestock population. For the zones which are not covered by the agricultural sample survey, an aerial survey was conducted in 2003. Livestock Development Master Plan Study (LDMPS) was also made an estimate in 2005/06 to incorporate all pastoral animals from administrative zones not sampled by CSA. These data sets were used to estimate the livestock population of the Somali region. However, hence these data are of 2005/06 the average national annual cattle, sheep and goats’ growth rate during 2006/07–2012/13 was used to compute the estimate for 2012/13. The whole Afar region was left out of this analysis because it is not cover by the census report of 2001/02.

After the district livestock share was computed based on 2012/13 agricultural sample survey report, Quantum GIS (Quantum GIS Development Team 2013) was used to join the respective districts’ cattle, sheep and goat population with district level administrative shape file obtained from DIVA-GIS. The district level cattle, sheep and goat population density per km^2^ was calculated by dividing the total cattle, sheep and goat population the district owns by the area in km^2^ of the district. In each district the cattle, sheep and goat population is assumed to be uniformly distributed. Here it is important to note that, there might be variations in livestock population densities among peasant associations within a given district which this study didn’t consider.

The proximity of livestock population to a given market infrastructure (major towns and all weather roads) was computed in QGIS using Zonal Statistics Plugin (Quantum GIS Development Team 2013). Before the proximity analysis was computed, the district level cattle, sheep and goat population density per km^2^ was created in raster layer (pixel size x = 1 km and y = 1 km). The country’s total land mass was classified into three zones. Zone one (areas within 10 km radius), zone two (areas between 11 to30 km radius) and zone three (areas greater than 30 km radius) from all-weather roads and major towns.

## Result and discussion

### Livestock population growth trend

**Figure 1:**
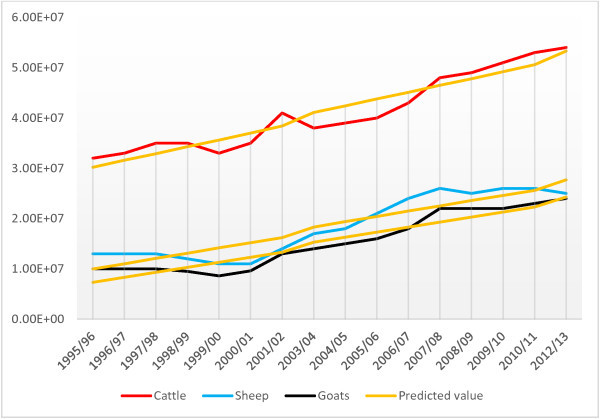
**Cattle, sheep and goat population growth trend (based on agricultural sample survey data).**

The study shows that the cattle and shoat population in sedentary areas of Ethiopia continues to grow. From 1995/96 to 2012/13 the cattle and shoat population grew from 54.5 million to over 103.5 million with average annual increment of 3.4 million. The livestock population of the country will continue to grow. In 2024/25 the cattle, sheep and goat population in sedentary areas of Ethiopia are estimated to reach 75, 42.8 and 39.6 million heads, respectively. The mid-year cattle, sheep and goat population of the sedentary part of the country from 1995/96 to 2012/13 is presented in Table 1. The cattle, sheep and goat population number increased significantly by time (Table 2). While the cattle population shows steady incline during this period, the sheep and goat population shows slower to declining growth from 1998 to 2001 and 2007 onwards (Figure 1). The regression analysis using regional dummies showed significant cattle and sheep population increases only for four regions namely Amhara, Oromia, SNNPR and Tigray Regional States. On the other hand, the goat population showed significant increase in all the regions except for Beneshangul Gumuz and Somali Regional States. The number of animals died increased significantly for all the three livestock species; however, the number of animals born increased significantly only for shoat (Table 2).

**Table 1:** **Livestock mid-year populations (agricultural sample survey), 1000 heads**

Year	Livestock species		
	Cattle	Sheep	Goats
1995/96	31,756	12,799	9,969
1996/97	33,083	13,465	10,413
1997/98	35,372	13,428	10,460
1998/99	35,095	12,236	9,544
1999/00	33,075	10,951	8,592
2000/01	35,383	11,438	9,621
2003/04	38,103	16,575	13,835
2004/05	38,749	18,075	14,859
2005/06	40,380	20,734	16,364
2006/07	43,007	23,617	18,423
2007/08	47,571	26,117	21,709
2008/09	49,298	25,017	21,884
2009/10	50,884	25,980	21,961
2010/11	53,382	25,509	22,787
2012/13	53,990	25,489	24,061

**Table 2:** **Univariate linear regression analysis**

Livestock species	Variables	Coefficient	95% CI	P-value
Cattle	Time period (year)	1355975	1108861 - 1603089	0.000
	Number of birth	0.139177	-1.48119 - 1.75954	0.856
	Number of death	1.483699	0.04384 - 2.92356	0.044
	Number slaughtered	-1.375457	-2.04224 - 0.70868	0.001
	Purpose			
	Milk	1.489767	0.40814 - 2.57139	0.011
	Beef	-1.240048	-2.32859 - 0.15150	0.030
	Draught	1.011168	0.36938 - 1.65296	0.007
	Breeding	1.484373	0.31096 - 2.65779	0.019
Sheep	Time period (year)	1638120	1279174 - 1997066	0.000
	Number of birth	1.5217	1.08769 - 1.95571	0.000
	Number of death	0.567583	-1.25985 - 2.39501	0.514
	Number slaughtered	1.625992	-.830245 - 4.08223	0.176
	Purpose			
	Mutton	-0.669687	-2.11112 - 0.77175	0.325
	Wool	-0.510576	-1.74252 - 0.72137	0.367
	Breeding	0.740964	0.54305 - 0.93889	0.000
Goat	Time period (year)	1065916	881464 - 1250367	0.000
	Number of birth	1.308132	1.07871 - 1.53755	0.000
	Number of death	2.482863	1.96793 - 2.99779	0.000
	Number slaughtered	-1.404435	-3.56936 - 0.76049	0.184
	Purpose			
	Milk	-0.091391	-2.05033 - 1.86755	0.921
	Meat	-0.480938	-2.25205 - 1.29017	0.549
	Breeding	0.900159	0.64326 - 1.15706	0.000

It is found that the number of cattle used for draught purpose increased significantly (Table 2). Furthermore, the shoat to cattle ratio tends to increase only in few regions as illustrated in Table 3. This is unexpected result because of ever increasing human population and land shortage. Their browsing behaviour, and minimum feed and water requirement is expected to induce greater sheep and goat production in the densely populated highlands as well as drought-prone pastoralist areas. However, in the highland areas of Ethiopia where crop production is dominant the farmer requires cattle for tillage. So, when herd size may have to be reduced due to land or feed shortage, farmers may prefer to retain cattle and give up some of the small ruminants. According to Jabbar et al. (2007), the combination of livestock species in a given situation depends not only on availability of feed resources but also on the function of the animals. This might be the reason why the shoat to cattle ratio didn’t increase in respective to the land shortage. Here it is important to note that this may not reflect the true picture of the lowland pastoral areas.

**Table 3:** **Shoat to cattle ratio in Ethiopia, 1995/96–2012/13**

Year	Tigray	Amhara	Benshangul Gumuz	Dire Dawa	Harari	Oromia	SNNPR
1995/96	1.34	0.87	0.95	3.25	0.87	0.58	0.61
1996/97	1.45	0.91	0.95	2.73	0.76	0.54	0.60
1997/98	1.04	0.83	0.81	2.30	0.65	0.52	0.52
1998/99	1.04	0.77	0.72	1.84	0.73	0.48	0.46
1999/00	0.90	0.71	0.71	1.77	0.78	0.48	0.49
2000/01	0.92	0.74	0.63	1.96	0.81	0.47	0.50
2001/02	0.92	0.87	0.84	2.31	0.73	0.49	0.66
2003/04	1.06	1.05	0.98	4.05	0.92	0.60	0.68
2004/05	1.04	1.08	1.01	4.09	0.96	0.68	0.70
2005/06	1.23	1.23	1.09	4.64	1.00	0.74	0.68
2006/07	1.27	1.28	1.33	4.77	1.03	0.77	0.78
2007/08	1.41	1.27	1.26	4.30	1.13	0.80	0.69
2008/09	1.45	1.18	0.99	3.38	0.92	0.74	0.70
2009/10	1.16	1.06	1.01	4.87	1.02	0.75	0.85
2010/11	1.19	1.04	1.10	5.72	0.97	0.71	0.69
2012/13	1.12	1.01	0.90	5.22	1.09	0.73	0.72

Livestock population estimates in Ethiopia are based on data collected from sedentary areas, which excludes large pastoral areas of Afar and Somali regions. According to Livestock Development Master Plan Study (LDMPS) figures for 2006, cattle in Afar and Somali regions constitute 8.2% of the national cattle, 34.6% of the national sheep, and 43.2% of the national goat population (Behnke 2010). Leaving out the pastoral livestock population from the trend analysis might have little influence on the livestock population growth trend; however, leaving out this portion of livestock population from GDP estimation could virtually underestimates contribution of livestock to the GDP. Accordingly, Behnke (2010) and latter Metaferia et al. (2011) have made a thorough review of the literature and analysis to come up with the real livestock population figure and contribution of the livestock subsector by incorporating the livestock population of the pastoral areas. The adjusted figure for cattle population is 3.2% greater than CSA data. Behnke (2010) estimate provide a considerably larger sheep and goat population, 40% and 59% higher than the CSA estimates. The main reason for which the agricultural sample survey report consistently underestimates the livestock population figures is that the survey have been confined to sedentary areas, excluding pastoral areas that have huge small ruminant population. These authors also estimated the real contribution of the livestock subsector to the national GDP. According to Metaferia et al. (2011), the GDP generated in the process of livestock production was estimated at birr 36.5 billion and GDP of livestock services in other sectors was estimated at birr 22.5 billion in 2008/09. This represents an increase of about 81% over the estimates made by Ministry of Finance and Economic Development (MOFED).

### Livestock distribution

**Figure 2:**
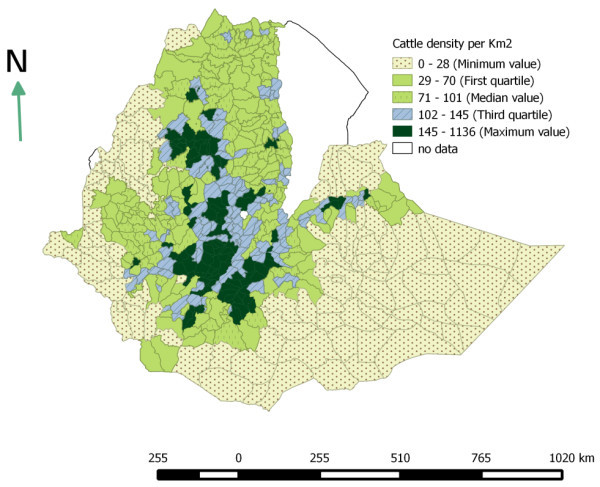
**Cattle population density per km** ^**2**^ **(based on Agricultural sample survey 2012/13).**

**Figure 3:**
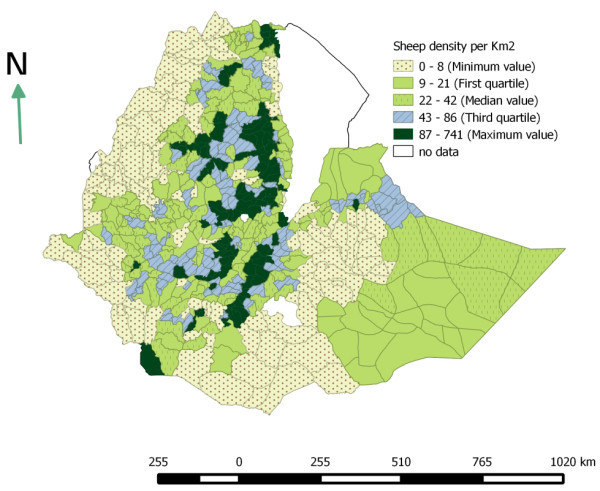
**Sheep population density per km** ^**2**^ **(based on Agricultural sample survey 2012/13).**

**Figure 4:**
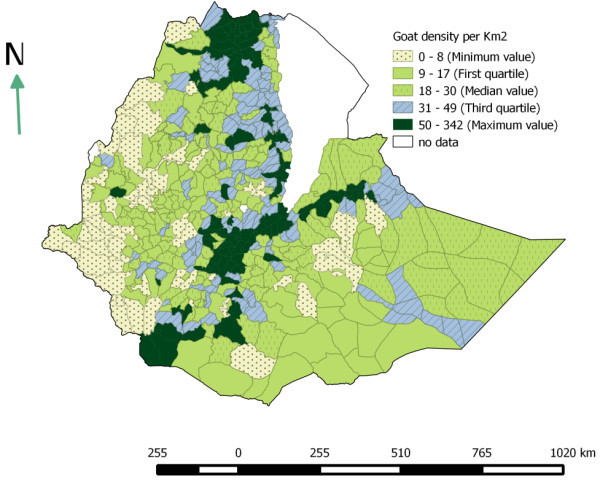
**Goat population density per km** ^**2**^ **(based on Agricultural sample survey 2012/13).**

The livestock population densities were computed as number of animals per km^2^ at district level. This study shows that cattle, sheep and goat population are unevenly distributed across space. The Ethiopian highlands are found to be highly populated. SNNPR, Amhara, Harari and Oromia are the highly populated region with respect to cattle population. High populations of sheep per km^2^ were found in Amhara, SNNPR and Dire Dawa. Dire Dawa, Harari and Tigray regions have the highest goat population per km^2^ (Table 4). On the other hand, the pastoral areas of Somali and Borena are found to have very low cattle density and relatively fair number of sheep and goat. The estimated cattle, sheep and goat densities per km^2^ are shown in Figures 2, 3 and 4.

**Table 4:** **Livestock population density per km** ^**2**^ **by region (based on agricultural sample survey 2012/13)**

Region	Livestock species		
	Cattle	Sheep	Goat
Afar	-	-	-
Addis Ababa	-	-	-
Amhara	116	85	36
Benshangul Gumuz	16	3	11
Dire dawa	44	63	167
Gambela	15	3	4
Harari	141	17	136
Oromia	111	43	32
SNNPR	147	83	53
Somali	7	24	25
Tigray	73	40	61

Key: (-) = no data.

In 2007 a study was conducted by Jabbar et al. (2007) to determine geographic distribution of livestock population in Ethiopia. The result found in this study is fairly similar with the finding of these authors. According to these authors, the highest numbers of cattle and shoats were found along a north–south transect covering parts of the central highlands of Tigray, Amhara and Oromia regions, and the transect that connects Adama and Dire Dawa.

Due to lack of data about the carrying capacity of the different areas, it is not possible to determine whether the figures reported in the maps are optimal or non-optimal. Generating data on factors which influence the carrying capacity will be obliging to determine the optimum carrying capacity cross space. Extent of available arable land, land-use, biomass productivity, farming system, and feeding system are the major factors which influence carrying capacity and stocking rate (Jabbar et al. 2007).

### Market access

**Figure 5:**
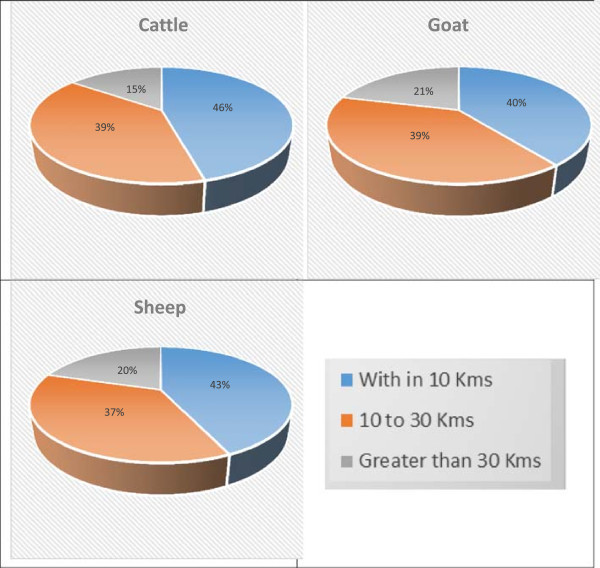
**Proportion of respective livestock species accessibility to all weather roads & major towns (based on Agricultural sample survey 2012/13).**

The proximity of cattle, sheep and goat population to a given market infrastructure is calculated using Zonal statistics. As shown in Figure 5, the highest numbers of livestock (46% of cattle, 43% of sheep and 40% goats) population were found to be located in zone one (within 10 km radius from the main towns and all-weather roads). These areas mainly cover the central highlands of Tigray, Amhara, Oromia and SNNPR regional states. This zone covers only 11% of the country’s landmass. On the other hand, 75% of the country’s land mass is located in zone three (greater than 30 km radius from market infrastructures). These areas are found to be not accessible to market infrastructure and support only 15, 20, and 21% of the country’s cattle, sheep and goat population respectively Figure 5.

**Figure 6:**
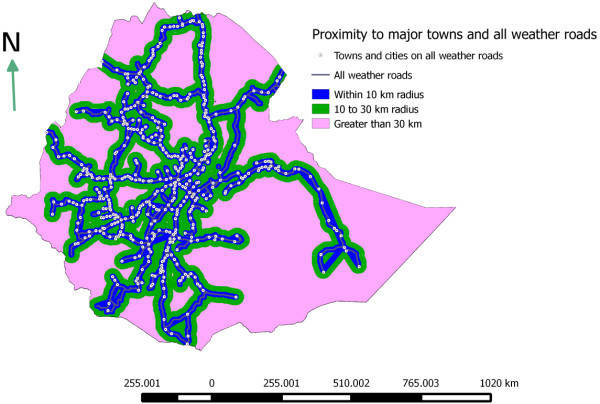
**Proximity to major livestock market center and all weather roads.**

In livestock and livestock products marketing road and market infrastructure is important to farmers, traders and consumers seeking to sell and/or buy livestock or livestock products. Underdevelopment and lack of market-oriented production, lack of adequate information on livestock resources and market information, inadequate marketing infrastructures, presence of trans-boundary animal diseases, and illegal trades are the major factors affecting livestock marketing in Ethiopia (Solomon et al. 2003). The study indicates that livestock production is positively correlated with infrastructure. As illustrated in Figure 6, the larger portion of the country landmass (75%) which has no or poor livestock marketing infrastructures is found to have a sparse livestock population.

In Ethiopia, trekking is widely used to take live animals from the production site to the primary and secondary market centers. In some parts of Somali, Oromia, and SNNP regions the pastoralists find markets after traveling 100-300 or more kilometers. Furthermore, almost all livestock trekking routes in the country are traditional and not facilitated with staging points where animals are provided rest, feed and water. As a result, animals are forced to travel longer distance without rest, feed, and water and this in turn resulted in weight loss, and even death (Dirbaba and Hurrissa 2009). Improving the market access in these remote areas is crucial in order to utilize the livestock resource of these areas as well as to boost the livestock production in these sparsely populated areas. The Ethiopian government is allocating a significant amount of money on infrastructural development especially on road development. Between 2004/05 – 2009/10 11,000 km all weather roads length was added to the existing network. As a result, the total all weather road length increased from 36,400 in 2004/05 to 48,000 km in 2009/10 (MOFED 2010). This indicates that in the coming years the proportion of livestock population accessible to market will significantly increase.

## Conclusion

The cattle, sheep and goat population of the country will continue to grow. The cattle and shoat population of the country are unevenly distributed across space. The Ethiopian highlands and areas which have a better infrastructure account for the largest share of livestock population. These areas are relatively more accessible to market infrastructure when compared to the pastoral lowland areas.

The livestock population estimates in Ethiopia are based on the data collected from the sedentary areas. The lowland pastoral areas of the country which exclusively practice livestock production are excluded from the estimates. The government of Ethiopia should look to an alternative means by which this portion of the livestock population of the country can be included to the estimates.
